# Quality of an Informed Consent Prior to a Surgical Intervention? Experience of a Teaching Hospital

**DOI:** 10.12669/pjms.321.8968

**Published:** 2016

**Authors:** Engin Kurt, Muharren Ucar, Adnan Atac

**Affiliations:** 1 Engin Kurt, MD, MBA, PhD, Assistant Professor, Department of Medical Ethics, Gulhane Medical Faculty, Ankara, Turkey; 2 MuharremUcar, MD, PhD, Associate Professor, Department of Medical Ethics, Gulhane Medical Faculty, Ankara, Turkey; 3 Adnan Atac, PhD, Professor, Department of Medical Ethics, Gulhane Medical Faculty, Ankara, Turkey

**Keywords:** Informed consent, Elucidation, Surgery, Medical Ethics

## Abstract

**Objective:**

To determine how far the information given by the physicians for the informed consent prior to the surgical intervention is comprehended by the patients.

**Methods:**

The study was carried out between July 1^st^, 2012 and July 1^st^, 2013 at Gulhane Medical Faculty. A total of 400 patients, who were in the third postoperative day after various surgical procedures (orthopedics, urology, ophthalmology, plastic surgery and breast surgery), were included in the study.

**Results:**

Of all the patients, 73.5% stated that the operative information was provided by physicians, whereas 22.7% claimed that no information was given in this regard. The patients who knew the name of the disease was 78%, while 18.3% did not know. Of all the patients, 25.7% knew the name of the operation, in contrast to of 52.3% who did not know it. About 12.5% of patients stated that they were not informed about the likely complications during the surgery, whereas 13.7% of patients reported that they were not informed about the post-operative complications.

**Conclusion:**

The verbal information and the written texts, different approaches such as drawings and visual materials (i.e. video’s and photographs) should be considered while providing information to the patients. While doing so the level of education of the community should also be taken into account.

## INTRODUCTION

Recently, the patient rights^1^, which take place in the third generation of human rights, have become more prominent in medical practice, and are guaranteed by the law, the legal regulations and the guidelines. These regulations have led the physicians to the necessity of informing the patients about likely complicaitons. The most important reflection of these legal regulations, which stem from the fundamental principles of medical ethics and are based on the principle of autonomy, is the “informed consent” applications in medical practice.

In clinical practice, important ethical problems arise when the informed consent is not obtained properly or is not obtained at all. Today, the negligence of obtaining a (proper) informed consent leads to probably the most commonly violated fundamental ethical principle with regard to the patients’ rights and physicians’ liability.^2^

This study was performed in a Gulhane Medical Faculty hospital on patients who underwent various surgical interventions and agreed to participate in the survey; in order to determine how far the information given by the physicians for the informed consent prior to the surgical intervention is comprehended by the patients, and to propose solutions for further improvement, if necessary.

## METHODS

This was a retrospective, descriptive study. A total of 435 patients who underwent surgical interventions in five different surgical disciplines (orthopedics, urology, ophthalmology, plastic surgery and breast surgery), were discharged from the intensive care to the in-patient clinics. On the third postoperative day; between July 1^st^, 2012 and July 1^st^, 2013 at Gulhane Medical Faculty they were given a questionnaire. However, 35 patients were excluded from the assessment due to the lack of credentials, double marking of answers in some questions and blank questions with a Likert scale. The Ethics Committee approval for the study was obtained before the commencement of the study.

A questionnaire consisting of 18 items was used. The questionnaire was prepared by the authors, considering the criteria for the national Patients’ Rights Directive and the Code of Conduct for Medical Profession, as well as the questionnaires used for similar studies in our country. The relationships of these variables were evaluated using the SPSS 19.0 statistical program. The consent of the illiterate patients was taken with the help of their companions. Of all the patients who participated in the study (n = 400); 80.8% (n = 323) were male, 60.2’s% (n = 241) were in the 20-29 age range, 40.7% (n = 163) were educated at secondary school level and 53.7% (n = 215) were single.

## RESULTS

The distribution of the socio-demographic characteristics of the patients is shown in Table-I. Of all the patients, 95.8% (n = 383) stated that he/she had signed a document to allow all kinds of treatments during the hospitalization, 73.5% (n = 294) stated that the information given about the surgery was provided by the physician, while 22.7% (n = 91) stated that no information was given at all.

**Table-I:** Socio-Demographic characteristics of the patients

	n	%
*Gender*		
Male	323	80.8
Female	77	19.2
Total	400	100
*Age*		
20-29	241	60.2
30-39	45	11.3
40-49	29	7.3
50-59	22	5.5
60 and above	63	15.7
*Education*		
University	122	30.5
Secondary Education	163	40.7
Primary education	83	20.7
Literate	17	4.3
Other	15	3.8
*Marital Status*		
Married	156	39.0
Single	215	53.7
Other	29	7.3

When the patients were asked to write the name of the disease, 18.3% (n = 73) did not know the name of the disease, and 52.3% (n = 209) did not know the name of the surgical intervention (Table-II).

**Table-II:** Answers to the questions of the patients participating in the study.

	n	%
*I signed a document to allow all kinds of treatments during the hospitalization*		
Yes	383	95.8
No	17	4.2
*Who informed you about your surgery?*		
Physician	294	73.5
Nurse	1	0.3
Trained Nurse	1	0.3
Other	5	1.2
Nobody gave information	91	22.7
Physician + Nurse	8	2.0
*Please write the name of your condition*		
Knows	312	78.0
Does not know	73	18.3
Does not know exactly	15	3.7
*Please write the name of the intervention*		
Knows	103	25.7
Does not know	209	52.3
Does not know exactly	88	22.0

Patients were asked 10 questions to assess the scope of consent. The highest rate of the most positive response (yes) was 82.8% (n = 331), to the questions “(1) I was given sufficient information about the diagnosis and the treatment of my condition after the hospitalization”, and “(2) I was given sufficient information and explanation about the diagnostic and the treatment methods”. The highest rate of the most negative response (no) was 19% (n = 76), to the question “I was informed about the likely duration of my surgery” (Table-III). The answers given to the questions by the patients participating in the study are shown in Table-III.

**Table-III:** The answers of the patients participating in the study in the scope of the informed consent.

	Yes		Partially Agree		No	

	n	%	n	%	n	%
I was given sufficient information about the diagnosis and the treatment of my condition after the hospitalization.	331	82.8	49	12.2	20	5.0
I was given sufficient information that my problem could be solved by surgical treatment (surgery).	331	82.8	56	14.0	13	3.2
I was given sufficient information about the alternative treatment options to surgery and the benefits and the risks.	307	76.8	65	16.2	28	7.0
I was given sufficient information about the time of the intervention.	281	70.3	43	10.7	76	19.0
I was given sufficient information about the benefits of this surgical intervention.	319	79.8	42	10.5	39	9.7
I was given sufficient information about the risks of this surgical intervention.	300	75	50	12.5	50	12.5
I was given sufficient information about the probable risks after this surgical intervention.	295	73.8	50	12.5	55	13.7
I was given sufficient information about the postoperative care.	320	80.0	38	9.5	42	10.5
I was given sufficient information about the length of hospital stay after this surgical intervention.	275	68.8	59	14.8	66	16.5
I was given sufficient information about the healing process after this surgical intervention.	288	72.0	48	12	64	16.0

## DISCUSSION

In the health care law, the surgical intervention becomes lawful by the informed consent. “Informed consent” provides the physician the right to perform some medical procedures on patient’s body.^3^ In many hospitals, usually during hospital admission and hospitalization, the patients themselves or their relatives have to sign a printed document in which “they declare that they accept all the medical intervention and treatment initiatives”. However, this official document they sign does not mean that an informed consent has been obtained.^3^ As Dubé-Baril has also expressed, “consent refers to a dialogue between the patient and the physician”, for this reason, the signature of the patient on a form does not mean that the physician has informed the patient sufficiently.^4^ Therefore, in order to be the document in question legally valid, the patient should be informed about his condition and the planned interventions in a manner that he can understand which also contains information concerning the matters according to the Patients’ Rights Directive and the Professional Ethics Rules of the Medical Association.^5^

In our study, 95.8% (n = 383) of the patients had a positive answer to the question “I signed a document to allow all kinds of treatments during the hospitalization”. This indicates that the pre-printed forms regarding informed consent is largely preferred.(Table-II). However, the fact that the patients sign this form with or without reading it does not mean that a consent process in compliance with medical ethics has taken place. Although such a signed form is accepted as evidence in a legal process, when the patient does not know how his condition is called, nor has adequate knowledge about the major surgical operation he has underwent, one cannot talk about a consent according to the medical ethics.

The education of the patients about the diagnosis and treatment of disease is physician’s responsibility. However, in our study, 73.5% (n = 294) of the patients stated that they were informed by the physician, while 22.7% (n = 91) stated that they were not informed at all. Deger et al. have reported that, 60% of the patients were informed by the physician who performed the surgery, whereas 23% was informed by a nurse.^7^ In a similar study on 106 patients, Siddiqui et al. have found that 8.5% of the patients were not informed.^8^ Most studies reveal that the information related to the intervention is usually given by the physicians. However, mostly the patients are not informed completely. A thorough explaination about the surgical procedures and the whole process by the physician who performs the surgery is expected both ethically and legally. Besides, the results of the treatment process have been observed to be more positive when the patient is informed adequately prior to surgery.^9^

Of all the patients; 18.3% (n = 73) did not know the name of the disease (diagnosis) at all, 52.3% (n = 209) did not know the name of the intervention at all; 3.7% (n = 15) did not know the name of the disease (diagnosis) exactly, and 22% (n = 88) did not know the name of the intervention exactly (Table-II). This can be explained by the fact that most patients had an educational level of secondary school. Cakir et al. have similarly found that the patients with a higher educational level were likely to be informed more thoroughly.^10^

The patients included in our study have generally accepted the way they were informed within the scope of informed consent positively. In this context, the information was most commonly given about “the possible diagnosis and treatment of the disease” and “the fact that the problem can be resolved by surgery”. The physicians have emphasized on two issues about which the patients worry most. This can be interpreted as an indicator of sufficient elucidation of the patients as mentioned in the Directory of Patient Rights^11^ and the Medical Ethics.

As to the level of knowledge of the patients; 1) It was determined that, 76.8% of patients (n = 307) were informed about the treatment options alternative to surgery, and their benefits and risks. On this issue, Ertem et al.^12^ and Turla et al.^3^ have reported a rate of 58.7% and 67%, respectively. 2) Regarding the risks during and after the surgery, 75% (n = 300) and 73.8% (n = 295) of the patients were informed, respectively. Ertem et al.^12^ had similar results, with a rate of 71.7%, whereas Siddiqui et al.^8^ have reported that, almost more than half of the patients were not informed about the risks of the surgery. It has also been reported that the patients who were not informed about the risks of the surgery regret after the intervention.^12^,^13^

In the literature as well as in our study, although it was found that the patients were explained about the probable complications during and after the surgery according to the Patient Rights and Medical Ethics, the presence of patients who were partially informed (in our study 12.5%, n=50) or who were not informed at all (in our study 12.5%, n=50) imply that the staff who is responsible for informing the patients, especially the physicians should be adequately trained.

About the post-operative care, the length of hospital stay and the recovery process, the patients were partially informed at a rate of 9.5%, 14.8% and 12%, and were not informed at all at a rate of 10.5%, 16.5%, and 16%, respectively. This indicates that the information concerning the postoperative period is given at a lower rate compared to that of the per-operative and the preoperative periods. Therefore, it can be stated that physicians are more susceptible to complications before and during the surgery, whereas it is not the case for the postoperative period.

However, there are studies reporting that as the patients are well-elucidated preoperatively about the circumstances of the postoperative period, their anxiety declines accordingly; furthermore, the subjective information before the surgery leads to an effective postoperative pain management.^14^ Studies show that patients are in need of being informed about the distress of surgery, the potential complications and the problems they might encounter in the healing process at home.^15^

## CONCLUSION

Scientific studies carried out indicate that proper information is a prerequisite for the patients to participate in the decision on their own health issues. However, our study reveals that there are inadequacies with regard to the elucidation of the patients. Although an informed consent with a complete education is difficult in practice, ideally a 100% explaination is obligatory. Therefore, it was concluded that for the surgical procedures to be adequate ethically and legally, the physicians need to be more precise about information and should give the explanations in a way that patients can understand. To achieve this, along with the oral information and the written texts, different approaches such as drawings and visual materials (i.e. video’s and photographs) should be used during which the level of education of the community should also be taken into account.
